# External Electric Field Effect on the Strength of σ-Hole Interactions: A Theoretical Perspective in Like⋯Like Carbon-Containing Complexes

**DOI:** 10.3390/molecules27092963

**Published:** 2022-05-05

**Authors:** Mahmoud A. A. Ibrahim, Nayra A. M. Moussa, Afnan A. K. Kamel, Mohammed N. I. Shehata, Muhammad Naeem Ahmed, Fouad Taha, Mohammed A. S. Abourehab, Ahmed M. Shawky, Eslam B. Elkaeed, Mahmoud E. S. Soliman

**Affiliations:** 1 Computational Chemistry Laboratory, Chemistry Department, Faculty of Science, Minia University, Minia 61519, Egypt; n.moussa@compchem.net (N.A.M.M.); a.kamel@compchem.net (A.A.K.K.); m.shehata@compchem.net (M.N.I.S.); 2 Department of Chemistry, The University of Azad Jammu and Kashmir, Muzaffarabad 13100, Pakistan; drnaeem@ajku.edu.pk; 3 Chemistry Department, Faculty of Science, Minia University, Minia 61519, Egypt; fouad.ahmed@mu.edu.eg; 4 Department of Pharmaceutics, Faculty of Pharmacy, Umm Al-Qura University, Makkah 21955, Saudi Arabia; maabourehab@uqu.edu.sa; 5 Science and Technology Unit (STU), Umm Al-Qura University, Makkah 21955, Saudi Arabia; amesmail@uqu.edu.sa; 6 Department of Pharmaceutical Sciences, College of Pharmacy, AlMaarefa University, Riyadh 13713, Saudi Arabia; ikaeed@mcst.edu.sa; 7 Molecular Modelling and Drug Design Research Group, School of Health Sciences, University of KwaZulu-Natal, Westville, Durban 4000, South Africa

**Keywords:** noncovalent interaction, σ-hole interactions, like⋯like complexes, EEF, SAPT

## Abstract

For the first time, σ-hole interactions within like⋯like carbon-containing complexes were investigated, in both the absence and presence of the external electric field (EEF). The effects of the directionality and strength of the utilized EEF were thoroughly unveiled in the (F-C-F_3_)_2_, (F-C-H_3_)_2_, and (H-C-F_3_)_2_ complexes. In the absence of the EEF, favorable interaction energies, with negative values, are denoted for the (F-C-F_3_)_2_ and (H-C-F_3_)_2_ complexes, whereas the (F-C-H_3_)_2_ complex exhibits unfavorable interactions. Remarkably, the strength of the applied EEF exhibits a prominent role in turning the repulsive forces within the latter complex into attractive ones. The symmetrical nature of the considered like⋯like carbon-containing complexes eradicated the effect of directionality of the EEF. The quantum theory of atoms in molecules (QTAIM), and the noncovalent interaction (NCI) index, ensured the occurrence of the attractive forces, and also outlined the substantial contributions of the three coplanar atoms to the total strength of the studied complexes. Symmetry-adapted perturbation theory (SAPT) results show the dispersion-driven nature of the interactions.

## 1. Introduction

Owing to their profuse contributions to various fields, including supermolecular chemistry [1,2,3], drug discovery [4,5,6], and molecular recognition [7,8,9], σ-hole interactions have recently attracted the interest of many experimentalists and theoreticians. The occurrence of σ-hole interactions was previously attributed to the presence of an electron-deficient region, compared to the surroundings, which is directly located along the extension of covalently bonded group IV–VII elements [10,11]. In addition, the σ-hole size is reported to be strongly associated with the polarizability of the σ-atom and the electron-withdrawing power of the attached group(s)/atom(s) [12,13]. The nomination of the σ-hole interactions to study was settled according to the position of the group IV–VII σ-hole bond donor in the periodic table as tetrel [14,15,16], pnicogen [13], chalcogen [17,18], and halogen [19,20,21] bonds.

Among the σ-hole interactions, diverse experimental [22,23,24] and theoretical [25,26,27] studies enticed significant scrutiny towards the tetrel bonding interactions, as a result of their primordial roles in catalytic chemistry [28,29] and ligand–protein interactions [24,30]. Further, the ability of tetrels to engage in like⋯like interactions was previously documented and precisely investigated [31,32,33], compared to the literature regarding pnicogen [34,35], chalcogen [36,37,38], and halogen [39,40,41]. The origin and nature of such interactions were widely demonstrated in the literature.

The efficacious role of the directionality and strength of the external electric field (EEF) on the intermolecular noncovalent interactions were precisely illustrated [42,43,44,45,46,47]. Initially, the EEF effect on Group VII σ-hole interactions was explained through tunning the Cl⋯N traditional halogen bond to a shared chlorine bond, or an ion-pair bond [48]. Subsequently, the elucidation of the EEF effect on the σ-hole interactions was expanded to involve group IV–VIII elements–Lewis base interactions [45,46,49]. Nevertheless, the impact of EEF on the σ-hole interactions within the like⋯like complexes is still ambiguous.

**Figure 1:**
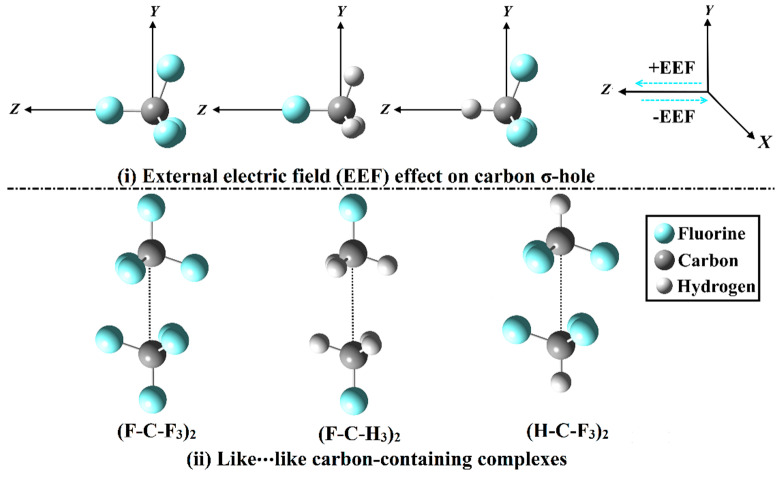
Representation of (**i**) the external electric field (EEF) effect on carbon σ-hole and (**ii**) the like⋯like carbon-containing complexes. Positive and negative signs represent the directionality of the employed EEF.

Accordingly, for the first time, σ-hole interactions within like⋯like carbon-containing complexes were delicately studied and comparatively explored, in both the absence and the presence of the positively- and negatively-directed EEF (Figure 1). Three carbon-containing complexes, namely, (F-C-F_3_)_2_, (F-C-H_3_)_2_, and (H-C-F_3_)_2_, were precisely investigated. A plethora of quantum mechanical calculations, including molecular electrostatic potential (MEP) and surface electrostatic potential extrema (*V*_s,max_), were performed for the considered monomers. Further, for the inspected like⋯like complexes, interaction energy, quantum theory of atoms in molecules (QTAIM), and noncovalent interaction (NCI) index analyses were executed. To pinpoint the physical nature of the investigated interactions, the symmetry-adapted perturbation theory (SAPT) analysis was adopted. The findings of this study provide versatile and noteworthy contributions to enhance the understanding of the effect of the EEF on the σ-hole interactions within like⋯like complexes.

## 2. Results and Discussion

### 2.1. MEP and V_s,max_ Calculations

**Figure 2:**
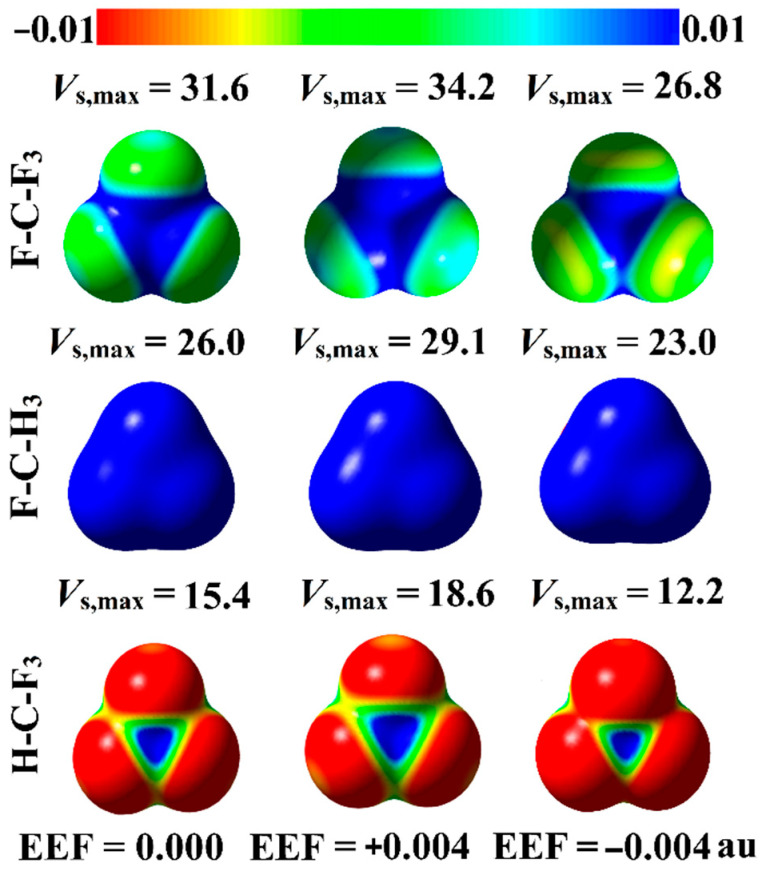
Molecular electrostatic potential (MEP) maps plotted at an 0.002 au electron density contour for F-C-F_3_, F-C-H_3_, and H-C-F_3_ optimized molecules in the absence of EEF (i.e., EEF = 0.000 au), and the presence of the positively- and negatively-directed EEF (i.e., +0.004 and −0.004 au, respectively). The electrostatic potential varies from −0.01 (red) to +0.01 (blue) au. The surface electrostatic potential extrema (*V*_s,max_) at the investigated σ-holes are given in kcal/mol.

**Figure 3:**
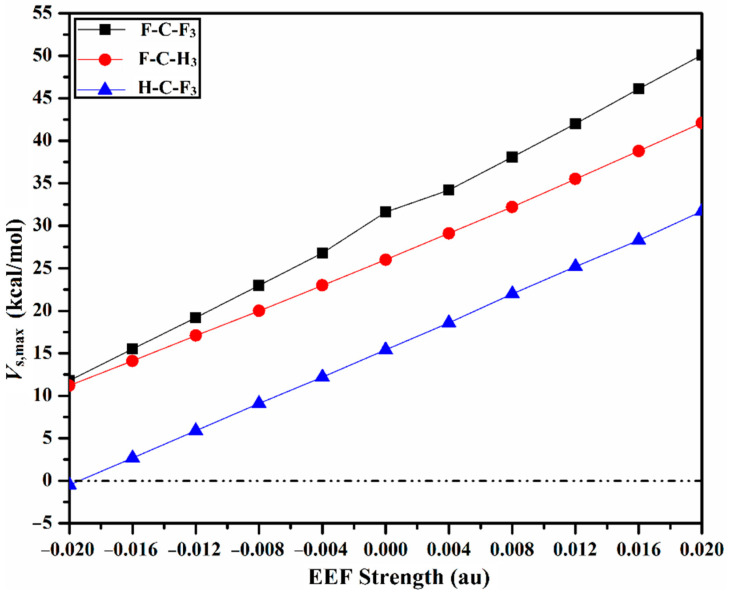
Correlation between the *V*_s,max_ values, and the magnitude of the external electric field (EEF). Positive and negative signs represent the directionality of the employed EEF.

Recent versatile studies demonstrate the molecular electrostatic potential (MEP) as a reliable technique to provide a powerful clue for the charge distribution over the molecular surface [50,51,52]. MEP maps were generated for the optimized carbon-containing molecules in the absence and the presence of the positively- and negatively-directed EEF, with values ranging from 0.004 to 0.020 au (Figures S1 and S2). Moreover, the surface electrostatic potential extrema (*V*_s,max_) was assessed in order to present quantitative evidence for the molecular electrostatic potential. MEP maps, along with *V*_s,max_ values, are depicted in Figure 2 for the optimized molecules in the absence of EEF (i.e., EEF = 0.000), and in the presence of +0.040 and −0.040 au EEF. The intercorrelation of the *V*_s,max_ values with the direction and magnitude of the applied EEF is represented in Figure 3.

From the MEP maps depicted in Figures S1 and S2, positive, blue-coded electrostatic potential regions (i.e., σ-hole) are detected along the outer surface of the carbon atoms, in varying sizes. As seen in Figure 2, the most prominent σ-hole size is found in the case of F-C-F_3,_ followed by F-C-H_3_ and H-C-F_3_, outlining the direct correlation between the σ-hole size and the electronegativity of the covalently bonded atoms to the central carbon atom. Numerically, in the absence of EEF, the *V*_s,max_ values increase as follows: H-C-F_3_ < F-C-H_3_ < F-C-F_3,_ with *V*_s,max_ values of 15.4, 26.0, and 31.6 kcal/mol, respectively.

Turning to the effect of EEF directionality, as illustrated in Figure 2, the sizes of the σ-holes increase and decrease by orienting the employed EEF in the positive and negative directions, respectively. In the same context, Figure 3 consistently reveals the direct and reverse correlation between the positive value of the surface electrostatic potentials extrema (*V*_s,max_), and the strength of the positively- and negatively-directed EEF, respectively.

Using the directional EEF, the σ-hole size exhibits a superior behavior (i.e., becomes more positive) by applying the EEF along the positive direction, whereas an inversed pattern is observed under the effect of the negatively-directed EEF. Illustratively, for the H-C-F_3_ molecule, the *V*_s,max_ value in the absence of EEF increases from 15.4 kcal/mol to 18.6 kcal/mol, and decreases from 15.4 kcal/mol to 12.2 kcal/mol, with the implementation of +0.004 au and −0.004 au EEF, respectively. Turning to the effect of EEF strength, direct and inverse correlations are disclosed between the σ-hole size and the magnitude of the positively- and negatively-directed EEF, respectively. For example, the *V*_s,max_ value of the H-C-F_3_ molecule increases to 18.6, 22.0, 25.2, 28.3, and 31.7 kcal/mol when the positively-directed EEF increases to 0.004, 0.008, 0.012, 0.016, and 0.020 au, respectively.

### 2.2. Interaction Energy

**Figure 4:**
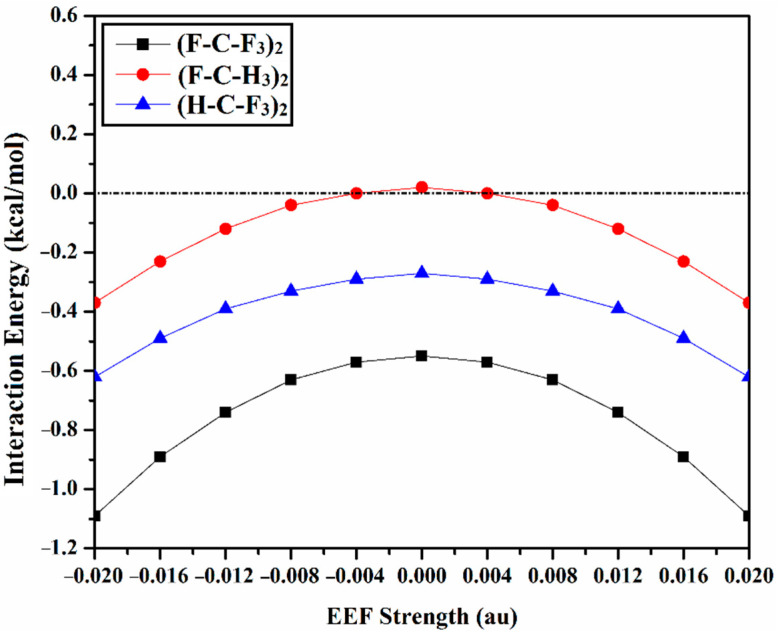
Interaction energy computed (in kcal/mol) at MP2/aug-cc-pVTZ level of theory for the optimized (F-C-F_3_)_2_, (F-C-H_3_)_2_, and (H-C-F_3_)_2_ complexes, in the absence and the presence of the positively- and negatively-directed external electric field (EEF), with values ranging from 0.004 to 0.020 au. Positive and negative signs represent the directionality of the employed EEF.

The σ-hole interactions of the like⋯like carbon-containing complexes were thoroughly studied in the absence and the presence of the EEF (see Figure 1). Initially, the (F-C-F_3_)_2_, (F-C-H_3_)_2_, and (H-C-F_3_)_2_ complexes were optimized at the MP2/aug-cc-pVTZ level of theory. For the optimized complexes, the interaction energies (*E*_1_) were evaluated at the same level of theory, and then benchmarked at the CCSD(T)/CBS level of theory (*E*_2_) (Table 1). The correlation between the interaction energies with the EEF direction and strength is graphically represented in Figure 4.

From data listed in Table 1, in the absence of the EEF (i.e., EEF = 0.000), the (F-C-F_3_)_2_ and (H-C-F_3_)_2_ complexes exhibit an elegant penchant to participate in noncovalent interactions, with negative interaction energies. On the other hand, positive values of interaction energies are denoted for the (F-C-H_3_)_2_ complex. This might be ascribed to the repulsive contributions of the three coplanar substituents. In general, interaction energies become less favorable according to the following order: (F-C-F_3_)_2_ > (H-C-F_3_)_2_ > (F-C-H_3_)_2_, with values of −0.55, −0.27, and 0.02 kcal/mol, respectively.

With regard to the effect of the EEF-based results, it is worth noting that the implementation of the positively- and negatively-directed EEF exhibit the same pattern on the considered like⋯like complexes (Figure 4). This similar amplitude could be ascribed to the domination of the symmetrical nature of the complexes under consideration, which led in turn to the elimination of the directionality effect of the applied EEF.

According to the data registered in Table 1, notable enhancement of the strength of the studied like⋯like complexes is observed when applying the positively- and negatively-directed EEF. For example, the MP2 energetic quantities of the optimized (F-C-F_3_)_2_ complexes are −0.57, −0.63, −0.74, −0.89, and −1.09 kcal/mol by implementing ±0.004, ±0.008, ±0.012, ±0.016, and ±0.020 au EEF, respectively.

The foregoing observations also highlight the role of EEF in turning the repulsive interactions (i.e., positive interaction energy) into attractive ones (i.e., negative interaction energy) within the studied complexes. Numerically, the MP2 interaction energies of the (F-C-H_3_)_2_ complex become more favorable, and turn from 0.02 kcal/mol (in the absence of EEF) to 0.00, −0.04, −0.12, −0.23, and −0.37 kcal/mol in the presence of ±0.004, ±0.008, ±0.012, ±0.016, and ±0.020 au EEF, respectively. Moreover, an inverse correlation is discerned between the C–C intermolecular distance and the strength of the applied EEF, alluding to the interaction energy enhancement.

Regarding energetic quantities, the MP2/aug-cc-PVTZ interaction energy is benchmarked at the CCSD(T)/CBS level of theory (Table 1), and both quantities are comparable, outlining a nearly similar trend.

### 2.3. QTAIM Analysis

**Figure 5:**
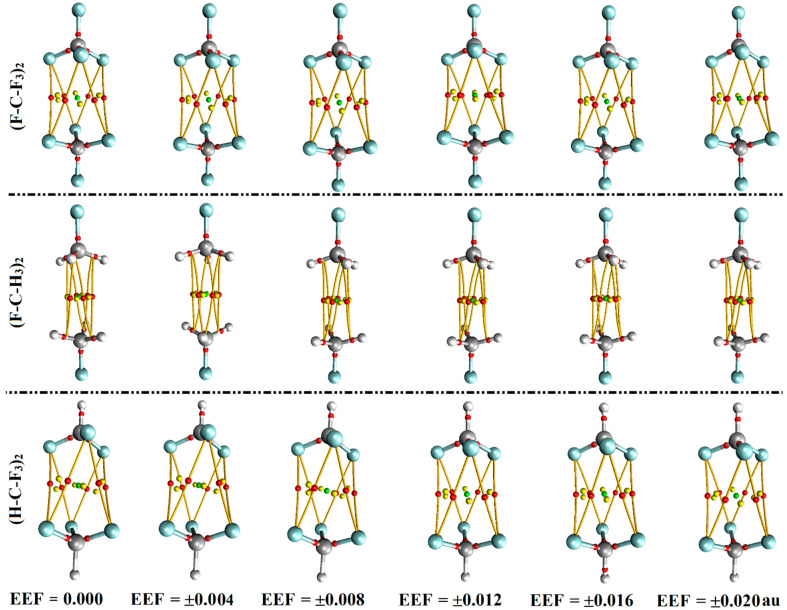
QTAIM diagrams of the optimized (F-C-F_3_)_2_, (F-C-H_3_)_2_, and (H-C-F_3_)_2_ complexes in the absence and the presence of the positively- and negatively-directed external electric field (EEF), with values ranging from 0.004 to 0.020 au. Positive and negative signs represent the directionality of the employed EEF. Red dots indicate the location of bond critical points (BCPs) and bond paths (BPs).

The quantum theory of atoms in molecules (QTAIM) was presented as an informative tool to precisely characterize both the origin and nature of the noncovalent interactions, based on the electron density features [53,54]. In the current study, QTAIM analysis was implemented to indicate the origin of the studied σ-hole interactions through generating the bond critical points (BCPs) and bond paths (BPs). Figure 5 displays the QTAIM diagrams for the considered complexes in the absence and the presence of the positively- and negatively-directed EEE. The topological parameters, including the electron density (*ρ*_b_), Laplacian (∇^2^*ρ*_b_), and total energy density (H_b_) were estimated and are collected in Table S1.

As illustrated in Figure 5, all the considered like⋯like complexes demonstrate six BCPs and BPs between the three coplanar substituents in each interacting monomer. These observations affirm the prominent contributions of the attractive forces between coplanar substituents over carbon analogs, which is in line with our earlier affirmations [31,55,56].

From Table S1, positive values are denoted for the electron density (*ρ*_b_), Laplacian (∇^2^*ρ*_b_), and total energy density (H_b_), ensuring the closed-shell nature of the investigated σ-hole interactions. Interestingly, the computed topological parameters highlight the EEF effect on the studied interactions, with growing *ρ*_b_, ∇^2^*ρ*_b_, and H_b_ values with an increasing EEF magnitude, which is in line with the energetic findings (Table S1). For instance, the electron density (*ρ*_b_) values of (F-C-F_3_)_2_ complex are 0.0029, 0.0029, 0.0033, 0.0034, and 0.0035 au, which exhibit interaction energies of −0.57, −0.63, −0.74, −0.89, and −1.09 kcal/mol in the presence of ±0.004, ±0.008, ±0.012, ±0.016, and ±0.020 au EEF, respectively.

### 2.4. NCI Analysis

The noncovalent interaction (NCI) index [45,57] was considered as a dependable index to characterize the noncovalent interactions, based on the electron density and its derivatives. The 3D NCI plots were generated for the (F-C-F_3_)_2_, (F-C-H_3_)_2_, and (H-C-F_3_)_2_ complexes using (λ_2_)*ρ* ranging from −0.035 (blue) to 0.020 (red), where the second eigenvalue of the hessian matrix and the electron density are represented by λ_2_ and *ρ*, respectively. Figure S3 shows the 3D NCI plots for the optimized complexes in the absence and the presence of the positively- and negatively-directed EEF. 

Based on the data displayed in Figure S3, the potentiality of the inspected carbon-containing molecules to participate in σ-hole interactions is assured and detected by the existence of green-colored isosurfaces between the two interacting molecules. Furthermore, the occurrence of circular-shaped green isosurface between the three coplanar substituents demonstrates their contributions, which is in line with the QTAIM affirmations. The superior effect of the EEF on the strength of the investigated complexes is evidently unveiled via increasing the green isosurfaces, by increasing the strength of the applied EEF.

### 2.5. SAPT Analysis

**Figure 6:**
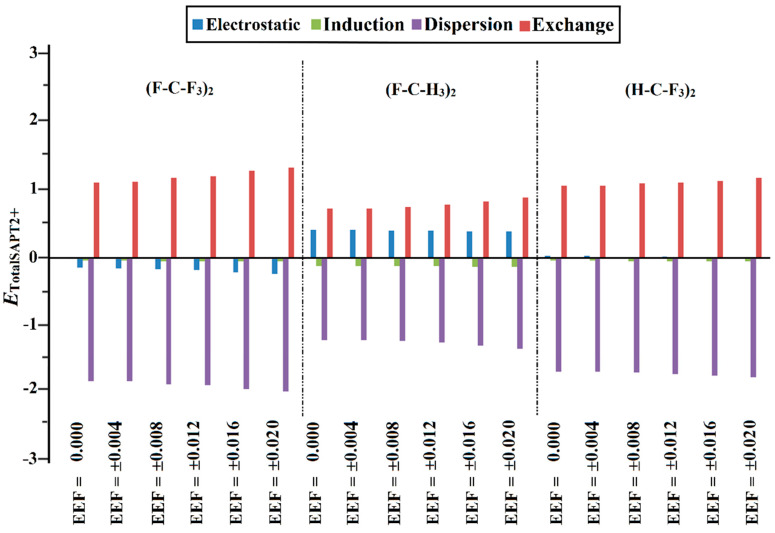
Bar chart illustrating physical components of SAPT2+ energy, including electrostatic (*E*_elst_), induction (*E*_ind_), dispersion (*E*_disp_), and exchange (*E*_exch_) terms of the optimized (F-C-F_3_)_2_, (F-C-H_3_)_2_, and (H-C-F_3_)_2_ complexes in the absence and the presence of the positively- and negatively-directed external electric field (EEF), with values ranging from 0.004 to 0.020 au. Positive and negative signs represent the directionality of the employed EEF.

Symmetry-adapted perturbation theory (SAPT) analysis was settled on as an authoritative tool to elucidate the physical forces beyond the occurrence of the noncovalent interactions [58]. SAPT was carried out for the (F-C-F_3_)_2_, (F-C-H_3_)_2_, and (H-C-F_3_)_2_ complexes at the SAPT2+ level of truncation (Figure 6). The total SAPT2+ energies, accompanied by the fundamental components of all the studied complexes, are compiled in Table S2.

For all the inspected complexes, the *E*_disp_ is found to be the most dominant force within the inspected interactions (Figure 6). In comparison, the contributions of the *E*_elst_ and *E*_ind,_ along with *E*_exch,_ are generally limited. Illustratively, the *E*_elst_, *E*_ind_, *E*_disp,_ and *E*_exch_ are −0.15, −0.04, −1.89, and 1.17 kcal/mol, respectively, in the case of the (F-C-F_3_)_2_ optimized complex in the absence of EEF (Table S2).

As listed in Table S2, the *E*_disp_ component exhibit the prevalent contributions of the studied complexes to the total energies in the absence and the presence of EEF. Notably, the contributions of *E*_disp_ are found to be enhanced in line with the interaction energy pattern as follows: (F-C-H_3_)_2_ < (H-C-F_3_)_2_ < (F-C-F_3_)_2._ For example, for (F-C-F_3_)_2_, (H-C-F_3_)_2_, and (F-C-H_3_)_2_, the *E*_disp_, calculated in the presence of ±0.020 au EEF, has values of −2.06, −1.84, and −1.39 kcal/mol, along with interaction energies of −1.09, −0.62, and −0.37 kcal/mol, respectively. Evidently, the favorable contributions of the *E*_elst_, *E*_ind_, and *E*_disp_ increase upon utilizing the positively- and negatively-directed EEF, which is in coincidence with the interaction energy findings (Table 1). For instance, the *E*_disp_ of the (F-C-F_3_)_2_ complex is −1.90, −1.94, −1.96, −2.02, and −2.06 kcal/mol upon applying an EEF of ±0.004, ±0.008, ±0.012, ±0.016, and ±0.020 au, respectively. The accuracy of the considered level for SAPT analysis is appreciated through assessing the energy difference between the MP2 energy and the computed SAPT2+ energy (ΔΔ*E*) (Table S2). The resulting tiny energy difference (ΔΔ*E*) outlines the accuracy of the utilized SAPT level of truncation.

## 3. Computational Methods

The inclination of carbon-containing molecules to engage in σ-hole interactions within like⋯like complexes is elucidated in the absence and the presence of the positively- and negatively-directed EEF (Figure 1). In the current study, (F-C-F_3_)_2_, (F-C-H_3_)_2_, and (H-C-F_3_)_2_ were chosen as the carbon-containing complexes. The EEF strength was employed with values ranging from 0.004 to 0.020 au, with an interval of 0.004 au. Geometrical optimization was executed at the MP2/aug-cc-pVTZ level of theory [59,60,61]. Molecular electrostatic potential (MEP) analysis was accomplished through generating MEP maps, and then assessing the surface electrostatic potential extrema (*V*_s,max_), using 0.002 au electron density contour. The value of electron density contour was selected to provide a precise characterization for the electrostatic potential on the molecular surfaces, as previously reported [62,63]. The extraction of the *V*_s,max_ values was also carried out using Multiwfn 3.7 software [64].

Within the optimized like⋯like complexes, the interaction energies were evaluated in the absence and the presence of the positively- and negatively-directed EEF, with values of 0.004, 0.008, 0.012, 0.016, and 0.020 au. The interaction energies were estimated as the difference in energy between the complex and the sum of the monomers. The benchmarking of the interaction energies was executed at the CCSD(T)/CBS level of theory [65,66], according to the idea that correlation energy is roughly proportional to X^−3^ for basis sets of the aug-cc-pVXZ type [67], using the following equations:

(1) $$E_{\mathrm{CCSD}\left(T\right)/\mathrm{CBS}\;}=\;\Delta E_{\mathrm{MP}2/\mathrm{CBS}}+\Delta E_{\mathrm{CCSD}\left(T\right)\;}$$

where:

(2) $$\Delta E_{\mathrm{MP}2/\mathrm{CBS}}=(64E_{\mathrm{MP}2/\mathrm{aug}-\mathrm{cc}-\mathrm{pVQZ}\;}-27E_{\mathrm{MP}2/\mathrm{aug}-\mathrm{cc}-\mathrm{pVTZ}})/37\;$$

(3) $$\Delta E_{\mathrm{CCSD}\left(T\right)\;}=E_{\mathrm{CCSD}\left(T\right)/\mathrm{aug}-\mathrm{cc}-\mathrm{pVDZ}\;}-E_{\mathrm{MP}2/\mathrm{aug}-\mathrm{cc}-\mathrm{pVDZ}}$$

By using the counterpoise (CP) correction procedure [68], the basis set superposition error (BSSE) was eradicated from the calculated interaction energies. The origin of the considered interactions was illustrated by generating the bond critical points (BCPs) and bond paths (PBs) with the utilization of the quantum theory of atoms in molecules (QTAIM) [61]. In the context of QTAIM, a variety of topological parameters, including electron density (*ρ*_b_), Laplacian (∇^2^*ρ*_b_), and total energy density (H_b_), were computed. NCI index analysis was also invoked, and the corresponding NCI plots were portrayed. The NCI isosurfaces were distinguished by the coloring scale of electron density (*ρ*) that distinguished the attractive forces (i.e., −0.035 au (blue)) from the repulsive ones (i.e., 0.020 au (red)) [69]. The QTAIM and NCI calculations were carried out via Multiwfn 3.7 software [64], and visualized using the Visual Molecular Dynamics (VMD) package [70]. All quantum mechanical calculations were performed using Gaussian 09 software [71].

Moreover, symmetry-adapted perturbation theory (SAPT) analysis was executed to reveal the physical nature of the σ-hole interactions. Using SAPT, the fundamental components, including the electrostatic (*E*_elst_), induction (*E*_ind_), dispersion (*E*_disp_), and exchange (*E*_exch_), were calculated for the studied complexes using the PSI4 code [72,73], at the SAPT2+ level of truncation. The sum of these physical components is given as follows [74]:

(4) $$E_{int}^{\mathrm{SAPT}2+}=E_{\mathrm{elst}}+E_{\mathrm{exch}}+E_{\mathrm{ind}}+E_{\mathrm{disp}}$$

where:

(5) $$E_{\mathrm{elst}}=E_{\mathrm{elst}}^{\left(10\right)}+E_{\mathrm{elst}}^{\left(12\right)}$$

(6) $$E_{\mathrm{ind}}=E_{\mathrm{ind},\mathrm{resp}}^{\left(20\right)}+E_{\mathrm{exch}-\mathrm{ind},\mathrm{resp}}^{\left(20\right)}+E_{\mathrm{ind}}^{\left(22\right)}+E_{\mathrm{exch}-\mathrm{ind}}^{\left(22\right)}+\;\delta E_{\mathrm{HF},\mathrm{resp}}^{\left(2\right)}$$

(7) $$E_{\mathrm{disp}}=E_{\mathrm{disp}}^{\left(20\right)}+E_{\mathrm{exch}-\mathrm{disp}}^{\left(20\right)}+E_{\mathrm{disp}}^{\left(21\right)}+E_{\mathrm{disp}\;}^{\left(22\right)}\left(\mathrm{SDQ}\right)+E_{\mathrm{disp}}^{\left(22\right)}T$$

(8) $$E_{\mathrm{exch}}=E_{\mathrm{exch}}^{\left(10\right)}+E_{\mathrm{exch}}^{\left(11\right)}+E_{\mathrm{exch}}^{\left(12\right)}$$

## 4. Conclusions

The predilection of carbon-containing molecules to engage in σ-hole interactions within the (F-C-F_3_)_2_, (F-C-H_3_)_2_, and (H-C-F_3_)_2_ complexes was inspected in the absence and the presence of the external electric field (EEF). In the absence of EEF, the MP2 energetic quantities addressed the occurrence of negative interaction energies for the (F-C-F_3_)_2_ and (H-C-F_3_)_2_ complexes with a higher favorability for the former. Upon the implementation of EEF along the positive and negative directions, the interaction energy escalates with the same magnitude, indicating the neglected effect of the EEF directionality on the strength of the like⋯like symmetrical complexes. In addition, the investigated like⋯like complexes demonstrate a supreme penchant to engage in favorable interactions when the applied EEF strength increases. The QTAIM results assert the closed-shell nature of the studied interactions. The SAPT calculations reveal the domination of the dispersion forces within all the studied complexes. These findings proclaim the prominent role of the EEF in enhancing the strength of the noncovalent interactions within like⋯like complexes, providing a fundamental linchpin for future studies related to crystal engineering and materials science.

## Figures and Tables

**Table 1:** Interaction energies calculated (in kcal/mol) at MP2/aug-cc-pVTZ (*E*_1_) and CCSD(T)/CBS (*E*_2_) levels of theory for the optimized (F-C-F_3_)_2_, (F-C-H_3_)_2_, and (H-C-F_3_)_2_ complexes in the absence and the presence of the positively- and negatively-directed external electric field (EEF), with values ranging from 0.004 to 0.020 au.

EEF ^a^ (au)	(F-C-F_3_)_2_			(F-C-H_3_)_2_			(H-C-F_3_)_2_		
	D ^b^	*E*_1_	*E*_2_	D ^b^	*E*_1_	*E*_2_	D ^b^	*E*_1_	*E*_2_
0.000	3.88	−0.55	−0.87	3.45	0.02	0.01	4.00	−0.27	−0.31
±0.004	3.88	−0.57	−0.89	3.45	0.00	−0.16	4.00	−0.29	−0.35
±0.008	3.87	−0.63	−1.00	3.44	−0.04	−0.18	4.00	−0.33	−0.42
±0.012	3.86	−0.74	−1.21	3.43	−0.12	−0.26	3.99	−0.39	−0.54
±0.016	3.84	−0.89	−1.58	3.41	−0.23	−0.38	3.98	−0.49	−0.73
±0.020	3.83	−1.09	−1.88	3.39	−0.37	−0.53	3.97	−0.62	−0.97

^a^ The positive and negative signs represent the directionality of the employed EEF. ^b^ Distances (D) computed (in Å) between the two carbon atoms in (F-C-F_3_)_2_, (F-C-H_3_)_2_, and (H-C-F_3_)_2_ complexes.

## Data Availability

Not applicable.

## Supplementary Materials

The following supporting information can be downloaded at: https://www.mdpi.com/article/10.3390/molecules27092963/s1, Figure S1. Molecular electrostatic potential (MEP) maps plotted at an 0.002 au electron density contour for F-C-F_3_, F-C-H_3_, and H-C-F_3_ optimized molecules in the presence of the positive EEF, ranged from +0.008 to +0.020; Figure S2. Molecular electrostatic potential (MEP) maps plotted at an 0.002 au electron density contour for F-C-F_3_, F-C-H_3_, and H-C-F_3_ optimized molecules in the presence of the negative EEF, ranged from −0.008 to −0.020; Figure S3. NCI plots of the optimized (F-C-F_3_)_2_, (F-C-H_3_)_2_, and (H-C-F_3_)_2_ complexes in the absence and the presence of the positively- and negatively-directed external electric field (EEF), with values ranging from 0.004 to 0.020 au; Table S1. Electron density (ρ_b_, au), Laplacian (∇^2^ρ_b_, au), and total energy density (H_b_, au) at bond critical points (BCPs) of the optimized (F-C-F_3_)_2_, (F-C-H_3_)_2_, and (H-C-F_3_)_2_ complexes in the absence and the presence of the positively- and negatively-directed external electric field (EEF), with values ranging from 0.004 to 0.020 au; Table S2. Electrostatic (*E*_elst_), induction (*E*_ind_), dispersion (*E*_disp_), exchange (*E*_exch_), and the estimated total SAPT energy (*E*_Total SAPT2+_) of the optimized (F-C-F_3_)_2_, (F-C-H_3_)_2_, and (H-C-F_3_)_2_ complexes in the absence and the presence of the positively- and negatively-directed external electric field (EEF), with values ranging from 0.004 to 0.020 au.

## References

[1] Priimagi A., Cavallo G., Metrangolo P., Resnati G. (2013). The halogen bond in the design of functional supramolecular materials: Recent advances. Acc. Chem. Res..

[2] Al-Hamdani Y.S., Tkatchenko A. (2019). Understanding non-covalent interactions in larger molecular complexes from first principles. J. Chem. Phys..

[3] Barrientos L., Miranda-Rojas S., Mendizabal F. (2019). Noncovalent interactions in inorganic supramolecular chemistry based in heavy metals. Quantum chemistry point of view. Int. J. Quantum Chem..

[4] Lu Y., Wang Y., Zhu W. (2010). Nonbonding interactions of organic halogens in biological systems: Implications for drug discovery and biomolecular design. Phys. Chem. Chem. Phys..

[5] Lu Y., Liu Y., Xu Z., Li H., Liu H., Zhu W. (2012). Halogen bonding for rational drug design and new drug discovery. Expert Opin. Drug Discov..

[6] Ibrahim M.A.A., Hasb A.A.M., Mekhemer G.A.H. (2018). Role and nature of halogen bonding in inhibitor···receptor complexes for drug discovery: Casein kinase-2 (CK2) inhibition as a case study. Theor. Chem. Acc..

[7] Fish R.H., Jaouen G. (2003). Bioorganometallic chemistry:  Structural diversity of organometallic complexes with bioligands and molecular recognition studies of several supramolecular hosts with biomolecules, alkali-metal ions, and organometallic pharmaceuticals. Organometallics.

[8] Mazik M. (2009). Molecular recognition of carbohydrates by acyclic receptors employing noncovalent interactions. Chem. Soc. Rev..

[9] Schalley C.A. (2000). Supramolecular chemistry goes gas phase: The mass spectrometric examination of noncovalent interactions in host–guest chemistry and molecular recognition. Int. J. Mass Spectrom..

[10] Clark T., Hennemann M., Murray J.S., Politzer P. (2007). Halogen bonding: The sigma-hole. Proceedings of “Modeling interactions in biomolecules II”, Prague, 5–9 September 2005. J. Mol. Model..

[11] Politzer P., Murray J.S., Clark T., Resnati G. (2017). The sigma-hole revisited. Phys. Chem. Chem. Phys..

[12] Scheiner S. (2017). Systematic elucidation of factors that influence the strength of tetrel bonds. J. Phys. Chem. A.

[13] Murray J.S., Lane P., Politzer P. (2007). A predicted new type of directional noncovalent interaction. Int. J. Quantum Chem..

[14] Grabowski S.J. (2014). Tetrel bond-sigma-hole bond as a preliminary stage of the SN2 reaction. Phys. Chem. Chem. Phys..

[15] Bauza A., Mooibroek T.J., Frontera A. (2013). Tetrel-bonding interaction: Rediscovered supramolecular force?. Angew. Chem. Int. Ed..

[16] Politzer P., Riley K.E., Bulat F.A., Murray J.S. (2012). Perspectives on halogen bonding and other σ-hole interactions: Lex parsimoniae (Occam’s Razor). Comput. Theor. Chem..

[17] Wang W., Ji B., Zhang Y. (2009). Chalcogen bond: A sister noncovalent bond to halogen bond. J. Phys. Chem. A.

[18] Murray J.S., Lane P., Clark T., Politzer P. (2007). Sigma-hole bonding: Molecules containing group VI atoms. J. Mol. Model..

[19] Cavallo G., Metrangolo P., Milani R., Pilati T., Priimagi A., Resnati G., Terraneo G. (2016). The halogen bond. Chem. Rev..

[20] Murray J.S., Concha M.C., Lane P., Hobza P., Politzer P. (2008). Blue shifts vs red shifts in sigma-hole bonding. J. Mol. Model..

[21] Politzer P., Murray J.S. (2013). Halogen bonding: An interim discussion. ChemPhysChem.

[22] Bauza A., Mooibroek T.J., Frontera A. (2016). Tetrel bonding interactions. Chem. Rec..

[23] Bauza A., Mooibroek T.J., Frontera A. (2014). Non-covalent sp(3) carbon bonding with ArCF3 is analogous to CH-pi interactions. Chem. Commun..

[24] Mahmoudi G., Bauza A., Amini M., Molins E., Mague J.T., Frontera A. (2016). On the importance of tetrel bonding interactions in lead(ii) complexes with (iso)nicotinohydrazide based ligands and several anions. Dalton Trans..

[25] Murray J.S., Lane P., Politzer P. (2009). Expansion of the sigma-hole concept. J. Mol. Model..

[26] Bauza A., Frontera A. (2015). Theoretical study on the dual behavior of XeO_3_ and XeF_4_ toward aromatic rings: Lone pair-π versus aerogen-π interactions. ChemPhysChem.

[27] Scheiner S. (2021). Competition between a tetrel and halogen bond to a common Lewis acid. J. Phys. Chem. A.

[28] Frontera A. (2020). Tetrel bonding interactions involving carbon at work: Recent advances in crystal engineering and catalysis. C J. Carbon Res..

[29] Karim A., Schulz N., Andersson H., Nekoueishahraki B., Carlsson A.C., Sarabi D., Valkonen A., Rissanen K., Grafenstein J., Keller S. (2018). Carbon’s three-center, four-electron tetrel bond, treated experimentally. J. Am. Chem. Soc..

[30] Mani D., Arunan E. (2014). The X–C···π (X = F, Cl, Br, CN) carbon bond. J. Phys. Chem. A.

[31] Ibrahim M.A.A., Moussa N.A.M., Soliman M.E.S., Moustafa M.F., Al-Fahemi J.H., El-Mageed H.R.A. (2021). On the potentiality of X-T-X3 compounds (T = C, Si, and Ge, and X = F, Cl, and Br) as tetrel- and halogen-bond donors. ACS Omega.

[32] Scheiner S. (2020). The ditetrel bond: Noncovalent bond between neutral tetrel atoms. Phys. Chem. Chem. Phys..

[33] Grabarz A., Michalczyk M., Zierkiewicz W., Scheiner S. (2020). Noncovalent bonds between tetrel atoms. ChemPhysChem.

[34] Esrafili M.D., Mohammadian-Sabet F. (2015). Pnicogen–pnicogen interactions in O_2_XP:PH_2_Y complexes (X = H, F, CN; Y = H, OH, OCH_3_, CH_3_, NH_2_). Chem. Phys. Lett..

[35] Setiawan D., Kraka E., Cremer D. (2015). Strength of the pnicogen bond in complexes involving group Va elements N, P, and As. J. Phys. Chem. A.

[36] Ibrahim M.A.A., Shehata M.N.I., Soliman M.E.S., Moustafa M.F., El-Mageed H.R.A., Moussa N.A.M. (2022). Unusual chalcogen···chalcogen interactions in like···like and unlike Y=C=Y···Y=C=Y complexes (Y = O, S, and Se). Phys. Chem. Chem. Phys..

[37] Li K., Li N., Yan N.N., Wang T.Y., Zhang Y.T., Song Q., Li H.J. (2020). Adsorption of small hydrocarbons on pristine, N-doped and vacancy graphene by DFT study. Appl. Surf. Sci..

[38] Sanchez-Sanz G., Trujillo C., Alkorta I., Elguero J. (2012). Intermolecular weak interactions in HTeXH dimers (X = O, S, Se, Te): Hydrogen bonds, chalcogen-chalcogen contacts and chiral discrimination. ChemPhysChem.

[39] Metrangolo P., Resnati G. (2014). Type II halogen···halogen contacts are halogen bonds. IUCrJ.

[40] Niyas M.A., Ramakrishnan R., Vijay V., Sebastian E., Hariharan M. (2019). Anomalous halogen-halogen interaction assists radial chromophoric assembly. J. Am. Chem. Soc..

[41] Ibrahim M.A.A., Moussa N.A.M. (2020). Unconventional type III halogen···halogen interactions: A quantum mechanical elucidation of σ-hole···σ-hole and di-σ-hole interactions. ACS Omega.

[42] Muruganathan M., Sun J., Imamura T., Mizuta H. (2015). Electrically tunable van der Waals interaction in graphene–molecule complex. Nano Lett..

[43] Arabi A.A., Matta C.F. (2011). Effects of external electric fields on double proton transfer kinetics in the formic acid dimer. Phys. Chem. Chem. Phys..

[44] Calvaresi M., Martinez R.V., Losilla N.S., Martinez J., Garcia R., Zerbetto F. (2010). Splitting CO_2_ with electric fields: A computational investigation. J. Phys. Chem. Lett..

[45] Ibrahim M.A.A., Saad S.M.A., Al-Fahemi J.H., Mekhemer G.A.H., Ahmed S.A., Shawky A.M., Moussa N.A.M. (2021). External electric field effects on the σ-hole and lone-pair hole interactions of group V elements: A comparative investigation. RSC Adv..

[46] Ibrahim M.A.A., Kamel A.A.K., Soliman M.E.S., Moustafa M.F., El-Mageed H.R.A., Taha F., Mohamed L.A., Moussa N.A.M. (2021). Effect of external electric field on tetrel bonding interactions in (FTF3···FH) complexes (T = C, Si, Ge, and Sn). ACS Omega.

[47] Foroutan-Nejad C., Marek R. (2014). Potential energy surface and binding energy in the presence of an external electric field: Modulation of anion–π interactions for graphene-based receptors. Phys. Chem. Chem. Phys..

[48] Xu H., Cheng J., Li Q., Li W. (2016). Some measures for making a traditional halogen bond be chlorine-shared or ion-pair one in FCl•NH3 complex. Mol. Phys..

[49] Ibrahim M.A.A., Mohamed Y.A.M., Abuelliel H.A.A., Rady A.S.S.M., Soliman M.E.S., Ahmed M.N., Mohamed L.A., Moussa N.A.M. (2021). σ-hole interactions of tetrahedral group IV–VIII lewis acid centers with lewis bases: A comparative study. ChemistrySelect.

[50] Weiner P.K., Langridge R., Blaney J.M., Schaefer R., Kollman P.A. (1982). Electrostatic potential molecular surfaces. Proc. Natl. Acad. Sci. USA.

[51] Politzer P., Laurence P.R., Jayasuriya K. (1985). Molecular electrostatic potentials: An effective tool for the elucidation of biochemical phenomena. Environ. Health Perspect..

[52] Murray J.S., Politzer P. (2011). The electrostatic potential: An overview. Wiley Interdiscip. Rev. Comput. Mol. Sci..

[53] Panini P., Gonnade R.G., Chopra D. (2016). Experimental and computational analysis of supramolecular motifs involving Csp2(aromatic)–F and CF3 groups in organic solids. New J. Chem..

[54] Bader R.F.W. (1985). Atoms in molecules. Acc. Chem. Res..

[55] Ibrahim M.A.A., Mahmoud A.H.M., Moussa N.A.M. (2020). Comparative investigation of ±σ–hole interactions of carbon-containing molecules with Lewis bases, acids and di-halogens. Chem. Pap..

[56] Ibrahim M.A.A., Ahmed O.A.M., Moussa N.A.M., El-Taher S., Moustafa H. (2019). Comparative investigation of interactions of hydrogen, halogen and tetrel bond donors with electron-rich and electron-deficient π-systems. RSC Adv..

[57] Contreras-Garcia J., Johnson E.R., Keinan S., Chaudret R., Piquemal J.P., Beratan D.N., Yang W. (2011). NCIPLOT: A program for plotting non-covalent interaction regions. J. Chem. Theory Comput..

[58] Jeziorski B., Moszynski R., Szalewicz K. (1994). Perturbation-theory approach to intermolecular potential-energy surfaces of van der Waals complexes. Chem. Rev..

[59] Møller C., Plesset M.S. (1934). Note on an approximation treatment for many-electron systems. Phys. Rev..

[60] Woon D.E., Dunning T.H. (1993). Gaussian basis sets for use in correlated molecular calculations. III. The atoms aluminum through argon. J. Chem. Phys..

[61] Woon D.E., Dunning T.H. (1994). Gaussian basis sets for use in correlated molecular calculations. IV. Calculation of static electrical response properties. J. Chem. Phys..

[62] Ibrahim M.A.A. (2012). Molecular mechanical perspective on halogen bonding. J. Mol. Model..

[63] Varadwaj P.R., Varadwaj A., Marques H.M. (2019). Halogen bonding: A halogen-centered noncovalent interaction yet to be understood. Inorganics.

[64] Lu T., Chen F. (2012). Multiwfn: A multifunctional wavefunction analyzer. J. Comput. Chem..

[65] Mishra B.K., Karthikeyan S., Ramanathan V. (2012). Tuning the C-H···Pi interaction by different substitutions in benzene-acetylene complexes. J. Chem. Theory Comput..

[66] Helgaker T., Klopper W., Koch H., Noga J. (1997). Basis-set convergence of correlated calculations on water. J. Chem. Phys..

[67] Nziko Vde P., Scheiner S. (2016). Comparison of pi-hole tetrel bonding with sigma-hole halogen bonds in complexes of XCN (X = F, Cl, Br, I) and NH3. Phys. Chem. Chem. Phys..

[68] Boys S.F., Bernardi F. (1970). The calculation of small molecular interactions by the differences of separate total energies. Some procedures with reduced errors. Mol. Phys..

[69] Johnson E.R., Keinan S., Mori-Sanchez P., Contreras-Garcia J., Cohen A.J., Yang W. (2010). Revealing noncovalent interactions. J. Am. Chem. Soc..

[70] Humphrey W., Dalke A., Schulten K. (1996). VMD: Visual molecular dynamics. J. Mol. Graph..

[71] Frisch M.J., Trucks G.W., Schlegel H.B., Scuseria G.E., Robb M.A., Cheeseman J.R., Scalmani G., Barone V., Mennucci B., Petersson G.A. (2009). Gaussian 09, Revision E01.

[72] Turney J.M., Simmonett A.C., Parrish R.M., Hohenstein E.G., Evangelista F.A., Fermann J.T., Mintz B.J., Burns L.A., Wilke J.J., Abrams M.L. (2012). PSI4: An open-source ab initio electronic structure program. Wiley Interdiscip. Rev. Comput. Mol. Sci..

[73] Hohenstein E.G., Sherrill C.D. (2010). Density fitting of intramonomer correlation effects in symmetry-adapted perturbation theory. J. Chem. Phys..

[74] Parker T.M., Burns L.A., Parrish R.M., Ryno A.G., Sherrill C.D. (2014). Levels of symmetry adapted perturbation theory (SAPT). I. Efficiency and performance for interaction energies. J. Chem. Phys..

