# Supplementary material for: External Electric Field Effect on the Strength of σ-Hole Interactions: A Theoretical Perspective in Like⋯Like Carbon-Containing Complexes
Source: Molecules. 2022 May 5;27(9):2963. doi: 10.3390/molecules27092963 (PMC9104924; doi:10.3390/molecules27092963)
Supplement: Supplementary file 1 [file molecules-27-02963-s001.zip › molecules-1692927-supplementary.pdf]

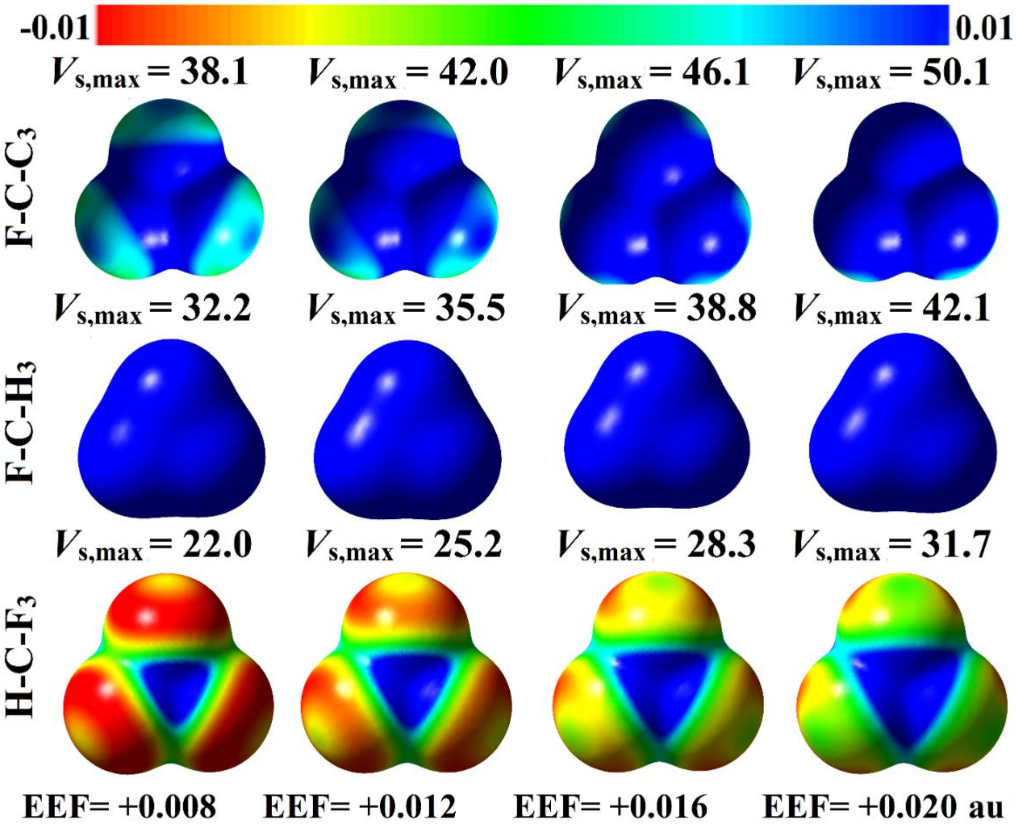

Figure S1. Molecular electrostatic potential (MEP) maps plotted at an 0.002 au electron density contour for F-C-F_3, F-C-H_3, and H-C-F_3 optimized molecules in the presence of the positive EEF ranged from +0.008 to +0.020). The electrostatic potential varied from −0.01 (red) to +0.01 (blue) au. The surface electrostatic potential extrema (Vs,max) at the investigated σ-holes are given in kcal/mol.

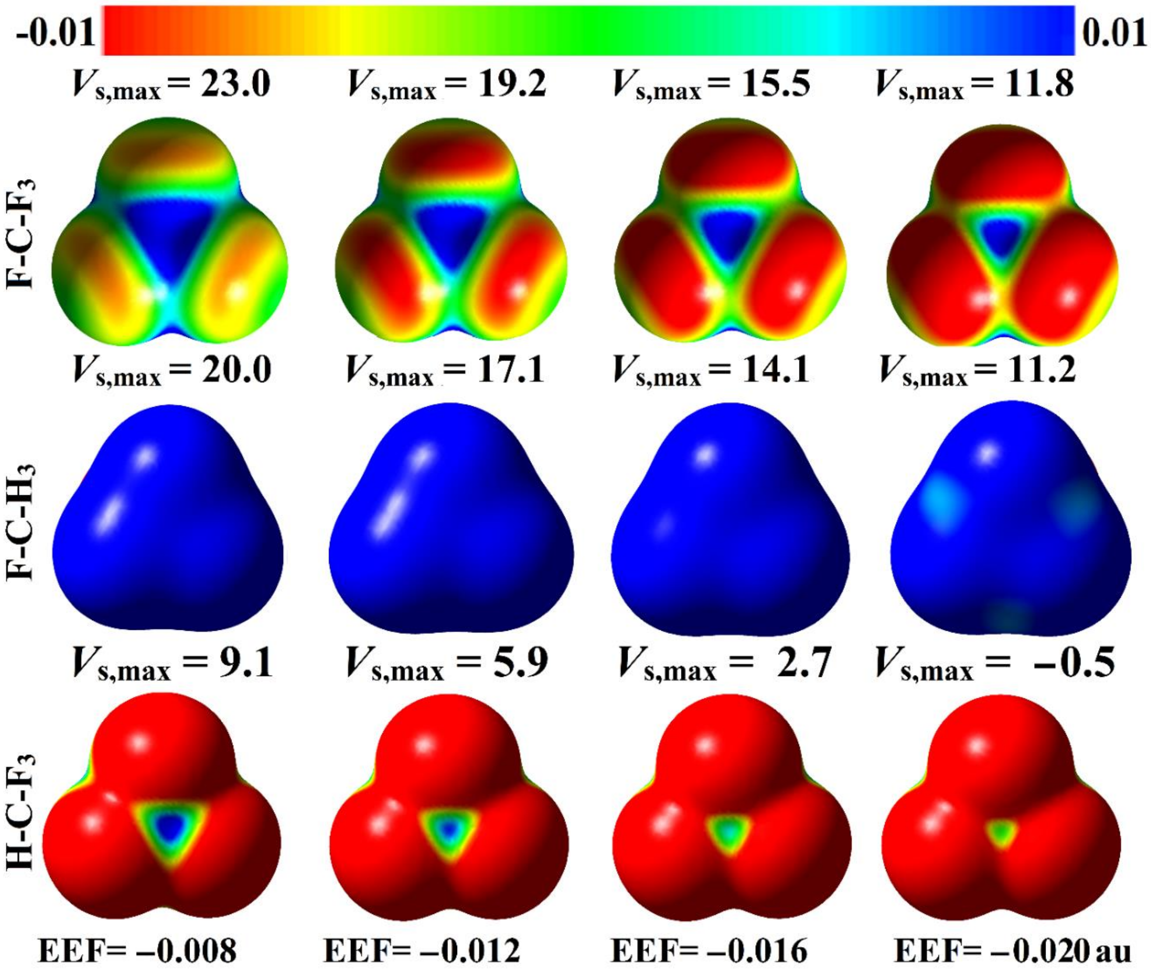

Figure S2. Molecular electrostatic potential (MEP) maps plotted at an 0.002 au electron density contour for F-C-F_3, F-C-H_3, and H-C-F_3 optimized molecules in the presence of the negative EEF ranged from –0.008 to –0.020). The electrostatic potential varied from −0.01 (red) to +0.01 (blue) au. The surface electrostatic potential extrema ($V_{s,max}$) at the investigated σ-holes are given in kcal/mol.

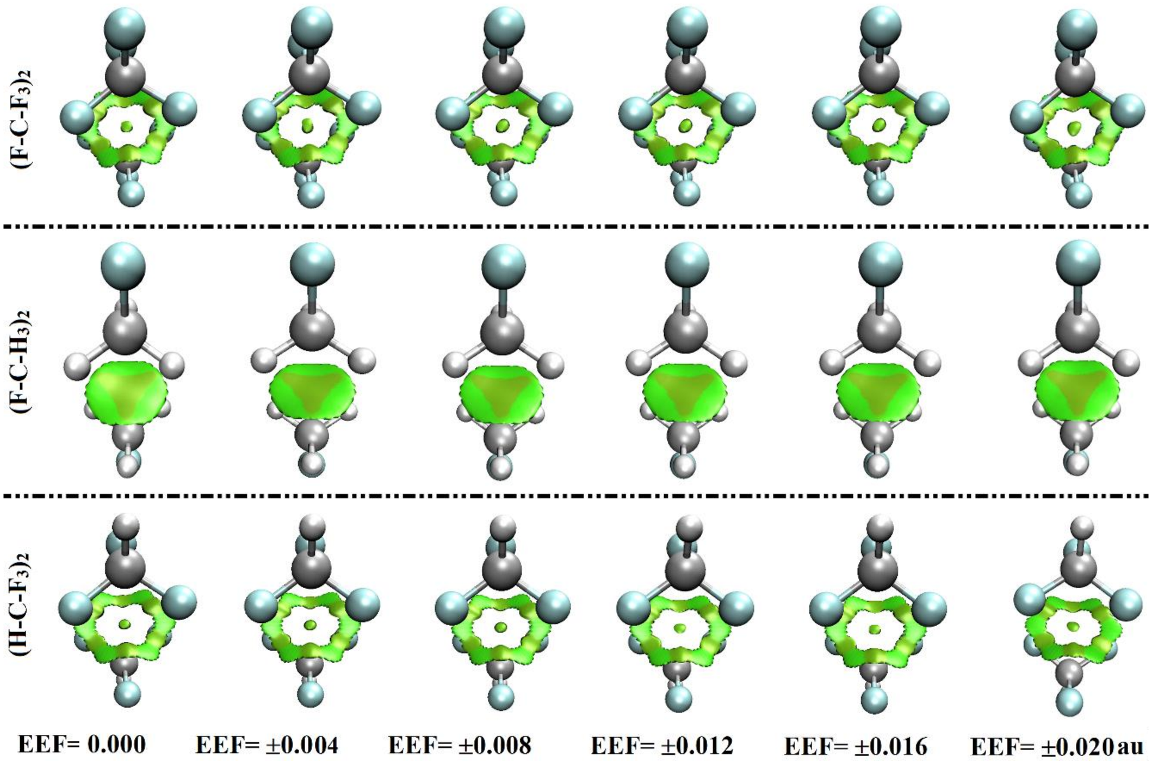

Figure S3. NCI plots of the optimized (F-C-F$_3$)$_2$, (F-C-H$_3$)$_2$, and (H-C-F$_3$)$_2$ complexes in the absence and the presence of the positively- and negatively-directed external electric field (EEF) with values ranging from 0.004 to 0.020 au. Positive and negative signs represent the directionality of the employed EEF. The utilized reduced density gradient of the isosurfaces is 0.50 au and colored from blue to red according to sign(λ_2)ρ ranging from −0.035 (blue) to 0.020 (red) au.

Table S1. Electron density (ρ_b, au), Laplacian ($\nabla^2\rho_b$, au), and total energy density (H_b, au) at bond critical points (BCPs) of the optimized $(F\text{-}C\text{-}F_3)_2$, $(F\text{-}C\text{-}H_3)_2$, and $(H\text{-}C\text{-}F_3)_2$ complexes in the absence and the presence of the positively- and negatively-directed external electric field (EEF) with values ranging from 0.004 to 0.020 au. Positive and negative signs represent the directionality of the employed EEF.

**EEF[a] (au)**	**$(F\text{-}C\text{-}F_3)_2$**		
	ρ_b (au)	$\nabla^2\rho_b$ (au)	H_b (au)
0.000	0.0028	0.0129	0.0006
±0.004	0.0029	0.0132	0.0006
±0.008	0.0029	0.0134	0.0006
±0.012	0.0033	0.0148	0.0006
±0.016	0.0034	0.0150	0.0007
±0.020	0.0035	0.0155	0.0007
**EEF[a] (au)**	**$(F\text{-}C\text{-}H_3)_2$**		
	ρ_b (au)	$\nabla^2\rho_b$ (au)	H_b (au)
0.000	0.0027	0.0136	0.0009
±0.004	0.0027	0.0137	0.0009
±0.008	0.0027	0.0139	0.0009
±0.012	0.0029	0.0147	0.0010
±0.016	0.0029	0.0148	0.0010
±0.020	0.0030	0.0153	0.0010
**EEF[a] (au)**	**$(H\text{-}C\text{-}F_3)_2$**		
	ρ_b (au)	$\nabla^2\rho_b$ (au)	H_b (au)
0.000	0.0026	0.0118	0.0006
±0.004	0.0026	0.0118	0.0006
±0.008	0.0026	0.0119	0.0006
±0.012	0.0028	0.0123	0.0006
±0.016	0.0028	0.0125	0.0006
±0.020	0.0029	0.0128	0.0006

[a] The positive and negative signs represent the directionality of the employed EEF.

Table S2. Electrostatic (E_{elst}), induction (E_{ind}), dispersion (E_{disp}), exchange (E_{exch}), and the estimated Total SAPT energy ($E_{TotalSAPT2+}$) of the optimized (F-C-F_3)$_2$, (F-C-H_3)$_2$, and (H-C-F_3)$_2$ complexes in the absence and the presence of the positively- and negatively-directed external electric field (EEF) with values ranging from 0.004 to 0.020 au. Positive and negative signs represent the directionality of the employed EEF.

**EEF[a] (au)**	**(F-C-F$_3$)$_2$**					
	E_{elst}	E_{ind}	E_{disp}	E_{exch}	$E_{TotalSAPT2+}$ [b]	$\Delta\Delta E$[c]
0.000	–0.15	–0.04	–1.89	1.17	–0.90	0.35
±0.004	–0.15	–0.04	–1.90	1.19	–0.90	0.33
±0.008	–0.17	–0.04	–1.94	1.25	–0.91	0.28
±0.012	–0.18	–0.04	–1.96	1.26	–0.92	0.18
±0.016	–0.21	–0.05	–2.02	1.35	–0.93	–0.10
±0.020	–0.24	–0.05	–2.06	1.40	–0.94	–0.15
**EEF[a] (au)**	**(F-C-H$_3$)$_2$**					
	E_{elst}	E_{ind}	E_{disp}	E_{exch}	$E_{TotalSAPT2+}$ [b]	$\Delta\Delta E$[c]
0.000	0.44	–0.11	–1.26	0.77	–0.16	0.18
±0.004	0.44	–0.11	–1.26	0.77	–0.16	0.16
±0.008	0.44	–0.12	–1.28	0.80	–0.16	0.12
±0.012	0.43	–0.12	–1.30	0.83	–0.16	0.04
±0.016	0.43	–0.13	–1.35	0.89	–0.17	–0.06
±0.020	0.42	–0.13	–1.39	0.95	–0.17	–0.20
**EEF[a] (au)**	**(H-C-F$_3$)$_2$**					
	E_{elst}	E_{ind}	E_{disp}	E_{exch}	$E_{TotalSAPT2+}$ [b]	$\Delta\Delta E$[c]
0.000	0.04	0.04	–1.75	1.12	–0.63	0.36
±0.004	0.04	0.04	–1.75	1.12	–0.63	0.34
±0.008	0.02	0.02	–1.76	1.15	–0.63	0.30
±0.012	0.03	0.03	–1.79	1.17	–0.63	0.24
±0.016	0.02	0.02	–1.81	1.20	–0.64	0.15
±0.020	0.00	0.00	–1.84	1.24	–0.64	0.02

[a] The Positive and negative signs represent the directionality of the employed EEF.

[b] $E_{\text{Total SAPT2+}} = E_{\text{elst}} + E_{\text{ind}} + E_{\text{disp}} + E_{\text{exch}}$.

[c] $\Delta\Delta E = E_{\text{MP2/aug-cc-pVTZ}} - E_{\text{SAPT2+}}$.
